# Case-based nuclear medicine

**DOI:** 10.1002/jmrs.9

**Published:** 2013-04-25

**Authors:** Geoffrey Paul Schembri

**Affiliations:** Department of Nuclear Medicine, Royal North Shore HospitalPacific Highway, St. Leonards, NSW, 2065, Australia

This second edition atlas presents 166 cases. Purchasing the book also entitles you to 12 months' access to further cases online.

The book is divided into 14 sections covering all the expected areas of imaging including 23 positron emission tomography/computed tomography (PET/CT) cases, 11 cardiac cases including single-photon emission computed tomography (SPECT) and PET, and a section on radioisotope therapy. The cases presented provide a good overview of commonly encountered conditions. The images are generally of good quality, although an occasional image lacks contrast. This does not prevent the relevant findings from being displayed and the teaching value of the case is not diminished.

The discussions are relevant and informative. While most experienced practitioners will already be aware of the issues, residents and registrars will find much to interest them. The authors end each case with a Pearls and Pitfalls section that is particularly useful to the doctor in training.

The technique used for each study is described in detail. Unnecessarily, this is repeated for each case even when identical to the last case. This is a US-based text. Doses are given in millicurie rather than standard SI units (becquerels).

Lung scanning cases remain well behind the forefront of current imaging techniques being restricted to planar images, often without a ventilation component. Xenon is still the main ventilation agent used, although one case with DTPA aerosol is present. No SPECT imaging or SPECT CT is evident. This section has little relevance to Australian trainees.

Similarly, the section on neuroendocrine scanning has no Ga68 DOTA cases and this radiopharmaceutical is not included in the discussion.

The online cases (currently 252) are a nice addition to the book.

Overall, I believe the book to be valuable and recommend it to those embarking on a nuclear medicine career. It covers most of the common indications and findings with helpful discussions and suggested further reading. Just skip the lung chapter!

